# Enhancing mental well-being of undergraduates: establishing cut-off values and analyzing substitutive effects of physical activity on depression regulation

**DOI:** 10.3389/fpsyg.2024.1432454

**Published:** 2024-09-10

**Authors:** Yue Ma, Yulin Gao, Hui Yang, Yu Zhang, Yixuan Ku

**Affiliations:** ^1^Faculty of Medicine, Macau University of Science and Technology, Taipa, Macau SAR, China; ^2^Philosophy and Social Science Laboratory of Reading and Development in Children and Adolescents (South China Normal University), Ministry of Education, Guangzhou, China; ^3^School of Nursing, Southern Medical University, Guangzhou, Guangdong, China; ^4^Guangdong Provincial Key Laboratory of Brain Function and Disease, Center for Brain and Mental Well-being, Department of Psychology, Sun Yat-sen University, Guangzhou, China

**Keywords:** subclinical depression, physical activity, sedentary, cut-off value, isochronous substitution

## Abstract

**Objective:**

This study aimed to analyze the effects of physical activity (PA), sleep quality, and sedentary behavior on subthreshold depression (StD) among undergraduates.

**Methods:**

This study included 834 undergraduates and assessed the impact of PA time, sleep quality, and sedentary behavior on depression. The receiver operating characteristic (ROC) analysis was performed to determine cut-off values for StD risk, while the isochronous substitution analysis was performed to evaluate the effects of different activities on depression regulation.

**Results:**

Gender, age, and academic grade had no significant influence on depression levels among undergraduates (*p* > 0.05). However, students engaging in sedentary behavior for more than 12.1 h per day or with a Pittsburgh Sleep Quality Index score above 3.5 were at an increased risk of subclinical depression. Additionally, the isochronous substitution of light-intensity physical activity for other activities (sleep, sedentary behavior, moderate and vigorous intensity physical activity) showed statistically significant effects (*p* < 0.05) in both 5-min and 10-min substitution models, demonstrating a positive effect on alleviating depression.

**Conclusion:**

The findings indicate that specific lifestyle factors, particularly high levels of sedentary behavior and poor sleep quality, are crucial determinants of subclinical depression among undergraduates, independent of demographic variables such as gender, age, and academic grade. Notably, light-intensity PA plays a key role in StD regulation, as substituting it with more intense physical activities or improving sleep quality substantially reduces depression scores. Furthermore, the benefits such substitution became more pronounced with the increase in duration of the activity.

## 1 Introduction

In the *Diagnostic and Statistical Manual of Mental Disorders*, Fifth Edition (DSM-5), a Beck Depression Inventory-II (BDI-II) score of 14 or higher with 2 major symptoms within 2 weeks is classified as subthreshold depression (StD) or subclinical depression (Rodríguez et al., 2012; Button et al., 2015). The prevalence of subclinical depression among undergraduates is alarmingly high, ranging from 13.2 to 39.1%, with a rapidly increasing trend (Bao et al., 2020). If left untreated, StD can lead to serious consequences, such as major depression and even suicide (Sheldon et al., 2021).

Several studies have shown that factors such as grade level, gender, and age may influence depression. For example, female students report significantly higher rates of depressive symptoms compared to male students (Acharya et al., 2018). Additionally, depressive symptoms tend to be more prevalent among older students (Bhandari et al., 2017). Other studies have identified age, grade level, and birthplace as influential factors (Wang et al., 2022). However, whether these factors influence StD remains unknown.

Undergraduates, who are in the early stages of adulthood, often experience abundant physical energy and frequent mood swings. They are also commonly confronted with challenges such as prolonged study hours, sedentary behavior (SB), and insufficient physical activity (IPA) (Li et al., 2021). SB, IPA, and sleep quality have been shown to significantly influence both survival indices and depression (Maddox et al., 2023). For example, the incidence of mild depression is four times higher in sedentary individuals compared to those who are physically active (Alotaibi and Boukelia, 2021). Engaging in more than 10 h of sedentary time per day is associated with a higher likelihood of depression (Schuch et al., 2020).

Physical activity (PA) has been proven to be an effective intervention for depression with minimal adverse events (Josefsson et al., 2014; Geneen et al., 2017). Some studies have demonstrated that engaging in PA reduces depression (Cooney et al., 2013), with 25–65 min of moderate PA (MPA) per day proving to be particularly effective (Chen et al., 2022). Supervised and group aerobic exercise programs, especially those conducted at a moderate intensity, have shown better outcomes (Heissel et al., 2023). However, few studies have analyzed the combined and quantitative effects of sleep and PA on StD in the undergraduate population.

Cut-off studies are often used to determine clinical parameter thresholds (Hajian-Tilaki, 2013; Nahm, 2022), which can help identify the PA and sleep indicators corresponding to the StD cut-off point (Negeri et al., 2021; Ghazisaeedi et al., 2022). The isochronous substitution analysis, which considers the 24-h day as a fixed component, encompassing sedentary time, moderate-to-vigorous PA (MVPA), light PA (LPA), and sleep time, reveals that an increase in one component necessitates a corresponding decrease in others (De Faria et al., 2022).

This study aims to provide a comprehensive quantitative analysis of how PA and sleep influence depression regulation among undergraduates. By defining the specific parameters that regulate depression through cut-off and substitution time analyses, this research offers valuable insights for undergraduates seeking to manage their depression and improve their sleep quality through tailored PA interventions.

## 2 Objects and methods

### 2.1 Objects

Between June 2022 and February 2024, using the PASS sample size calculation tool, this study determined the required sample size to be 513 participants; considering a 10% attrition rate, this resulted in a final total sample size of 570.

Participants were eligible for inclusion if they met the following criteria: (1) undergraduate students (not taking a break from school); (2) healthy enough to perform daily activities. The exclusion criteria are as follows (1) an existing diagnosis of a mental illness; (2) use of psychotropic medications, and (3) refusal to participate in the study. All participants provided their written informed consent after receiving a detailed explanation of the study, which was conducted in accordance with the Declaration of Helsinki.

The study was approved by the Ethics Committee of Southern Medical University[2023(NO.25)]. For the final analysis, 834 participants were randomly selected from each grade level at a university in China to complete an online questionnaire.

### 2.2 Questionnaires

#### 2.2.1 International Physical Activity Questionnaire (IPAQ)

This study used the long form of the International Physical Activity Questionnaire (IPAQ), which consists of 21 questions addressing physical activities related to work, transportation, household chores and gardening, leisure sports, and sedentary time (Schuch et al., 2021). The reliability and validity of the Chinese version of the IPAQ were verified using a repeated survey method, which showed a correlation coefficient of 0.94 between the two sets of scores, indicating good test–retest reliability and construct validity (Liang et al., 2010). This study specifically focused on the time spent in various intensities of PA and the total sedentary time (Fan et al., 2014).

#### 2.2.2 Beck Depression Inventory-II (BDI-II)

The Beck Depression Inventory comprises 21 items and is widely regarded as the gold standard for self-assessment of depression (Dozois et al., 1998). It employs a 4-point scoring system (0–3), with a total score ranging from 0 to 63 points (Kaya et al., 2021). The Cronbach’s α coefficient for the Chinese version of the BDI-II is 0.94 (Wang et al., 2011).

Based on DSM-5 criteria, this study excluded individuals diagnosed with depression. For the purpose of classification, a BDI-II score greater than 14, along with the presence of two major symptoms within 2 weeks, was categorized as subclinical depression (Takagaki et al., 2014; Spek et al., 2008; Zhang et al., 2021).

#### 2.2.3 Pittsburgh Sleep Quality Index (PSQI)

The Pittsburgh Sleep Quality Index is a self-report questionnaire that assesses sleep quality. It consists of 19 self-rated items and 5 items rated by others, categorized into seven components: A. sleep quality, B. time to fall asleep, C. sleep duration, D. sleep efficiency, E. sleep disturbances, F. use of sleeping medication, and G. daytime dysfunction. The total PSQI score ranges from 0 to 21, with higher scores indicating poorer sleep quality. The Cronbach’s α coefficient for the Chinese version of the PSQI is 0.89 (Liu et al., 1996), indicating good internal consistency (Fawzy and Hamed, 2017; Liu et al., 2021).

### 2.3 Statistical methods

General information was analyzed using IBM SPSS 20.0 to examine potential differences in depression scores by gender and grade level. Correlation and regression analyses were conducted, incorporating ROC cutoff values (Dolle et al., 2012), to analyze the effects of physical activity (PA) and sleep indices on depression. The isochronic substitution analysis was performed using R (version 3.6.3) (Dumuid et al., 2019) by selecting four indicators—sedentary time, light physical activity (LPA) time, moderate-to-vigorous physical activity (MVPA) time, and sleep time—to explore the effects of substituting time spent in different activities on reducing depressive moods (Pasanen et al., 2022). The results are reported using 5-min and 10-min isochronous substitution models (Dumuid et al., 2019).

## 3 Results

### 3.1 Demographic analysis of depression scores

In the analysis of basic demographic variables, no statistically significant differences in depression scores were found based on gender, age, or academic grade (see Table 1).

**Table 1:** Univariate associations of demographic characteristics with BDI scores (*n* = 834).

Items		(*n*)	(%)	BDI-II (Means±SD)	*r/t/F*	*p*
Age	Average age	21.41 ± 1.65		8.97 ± 8.32	*r* = 0.03	0.43
Gender	Men	123	14.70	10.07 ± 10.12	*t* = 1.34	0.18
	Women	711	85.30	8.78 ± 7.69		
Grade	Freshman	197	23.62	9.76 ± 8.64	*F* = 2.19	0.09
	Sophomore	216	25.90	9.44 ± 8.80		
	Junior	223	26.74	7.84 ± 7.39		
	Senior	198	23.74	8.95 ± 8.37		

### 3.2 The association between physical activity, sedentary behavior, sleep, and StD

**Figure 1:**
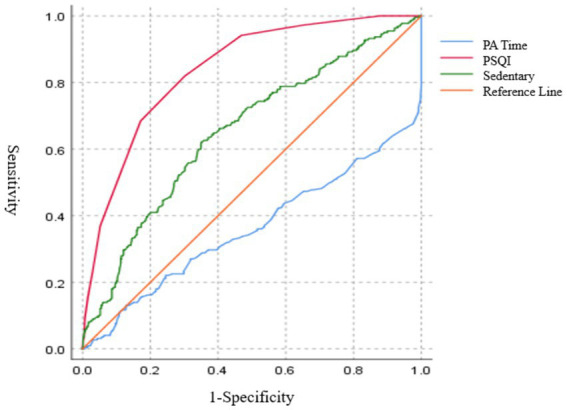
ROC curves for sleep quality, sedentary time, and PA time on StD risk.

Furthermore, the regression analysis highlighted the significant effect of PA time, sleep quality, and sedentary time on depression (Table 2). The ROC curve analysis, used to determine cutoff values, is shown in Figure 1.

**Table 2:** Binary logistic regression model outcomes for StD risk (*n* = 834).

Items	B	SE	*p*	*Wald*	Exp B	95% CI for Exp B	
						Lower	Upper
Constant	−5.25	1.22	<0.01	18.55	0.01		
PA time	−0.2	0.09	0.03	4.64	0.82	0.69	0.98
PSQI	0.58	0.05	<0.01	155.66	1.78	1.62	1.95
Sedentary time	0.2	0.09	0.02	5.09	1.22	1.03	1.45

Sedentary time over 12.1 h per day and a sleep quality score greater than 3.5 increased the risk of StD (see Table 3).

**Table 3:** ROC analysis and cutoff values for StD.

Items	AUC	SE	*p*	Cut-off values	Sensitivity	Specificity	Youden index
PA time	0.36	0.02	<0.01	4.49 h	0.13	0.87	<0.01
Sedentary time	0.65	0.02	<0.01	12.10 h	0.62	0.65	0.27
PSQI	0.84	0.15	<0.01	3.50	0.82	0.70	0.52

AUC, Area under curve.

### 3.3 Substitution analysis of physical activity

Although some studies have indicated that a minimum duration of 5 min can be effective for certain health benefits (Su, 2022), 10 min is often considered the smallest unit for individual health benefits and the shortest duration at which sedentary behavior poses a health risk (Li et al., 2020). Therefore, in this study, the substitution analysis was conducted for both 5-min and 10-min intervals. Moderate and vigorous physical activities were combined as MVPA. As shown in Table 4, an increase in LPA by 10 min accompanied by a reduction in sedentary time or engaging in MVPA is associated with a significant decrease in depression.

**Table 4:** Component isochronous substitution analysis of physical activity and BDI-II.

Time	(+Δ)	(−Δ)	*β*	95%CI (low, up)	*p*
5 min	(+Δ) Sleep	(−Δ) Sedentary	−0.16	(−0.23, −0.10)	<0.01
	(+Δ) Sleep	(−Δ) LPA	0.30	(0.18, 0.41)	<0.01
	(+Δ) Sleep	(−Δ) MVPA	−0.27	(−0.36, −0.17)	<0.01
	(+Δ) Sedentary	(−Δ) Sleep	0.16	(0.10, 0.23)	<0.01
	(+Δ) Sedentary	(−Δ) LPA	0.46	(0.36, 0.56)	<0.01
	(+Δ) Sedentary	(−Δ) MVPA	−0.11	(−0.19, −0.02)	0.01
	(+Δ) LPA	(−Δ) Sleep	−0.24	(−0.34, −0.14)	<0.01
	(+Δ) LPA	(−Δ) Sedentary	−0.40	(−0.48, −0.32)	<0.01
	(+Δ) LPA	(−Δ) MVPA	−0.51	(−0.63, −0.38)	<0.01
	(+Δ) MVPA	(−Δ) Sleep	0.25	(0.16, 0.34)	<0.01
	(+Δ) MVPA	(−Δ) Sedentary	0.09	(0.01, 0.16)	0.02
	(+Δ) MVPA	(−Δ) LPA	0.55	(0.42, 0.68)	<0.01
10 min	(+Δ) Sleep	(−Δ) Sedentary	−0.32	(−0.45, −0.20)	<0.01
	(+Δ) Sleep	(−Δ) LPA	0.69	(0.44, 0.94)	<0.01
	(+Δ) Sleep	(−Δ) MVPA	−0.57	(−0.77, −0.36)	<0.01
	(+Δ) Sedentary	(−Δ) Sleep	0.33	(0.20, 0.45)	<0.01
	(+Δ) Sedentary	(−Δ) LPA	1.01	(0.79, 1.23)	<0.01
	(+Δ) Sedentary	(−Δ) MVPA	−0.24	(−0.43, −0.06)	0.01
	(+Δ) LPA	(−Δ) Sleep	−0.43	(−0.62, −0.24)	<0.01
	(+Δ) LPA	(−Δ) Sedentary	−0.76	(−0.91, −0.60)	<0.01
	(+Δ) LPA	(−Δ) MVPA	−1.00	(−1.25, −0.75)	<0.01
	(+Δ) MVPA	(−Δ) Sleep	0.48	(0.32, 0.65)	<0.01
	(+Δ) MVPA	(−Δ) Sedentary	0.15	(0.01, 0.30)	0.04
	(+Δ) MVPA	(−Δ) LPA	1.16	(0.89, 1.44)	<0.01

## 4 Discussion

### 4.1 Prolonged sedentary behavior and poor sleep quality as indicators of subthreshold depression risk

This study found that a PSQI score above 3.5 and more than 12 h of sedentary behavior per day significantly increase the risk of StD. These findings align with previous research that highlights the impact of sleep quality on depression and StD (Gardani et al., 2022), emphasizing the urgent need to enhance students’ awareness about the critical thresholds for maintaining sleep quality.

Regarding sedentary behavior, previous research has indicated that 8.5–10 h of sedentary time is associated with increased all-cause mortality and depression (Ku et al., 2019), which is notably lower than the threshold identified in this study. This discrepancy may be due to several factors. First, the demographic composition of this study, mainly consisting of undergraduate students, may exhibit a higher tolerance to the adverse effects of sedentary behavior compared to older adults, possibly due to their relatively faster metabolism. Additionally, existing research has suggested a dose–response relationship between PA and depression, with higher PA levels potentially exacerbating depression symptoms (Pearce et al., 2022). Furthermore, differences in sedentary time observed between this study and previous literature could result from methodological variations in activity measurement, such as the use of different instruments (Schmid et al., 2015; Ku et al., 2019).

Therefore, this study emphasizes the need for moderation in PA engagement while balancing sedentary behavior and sleep, offering nuanced insights for promoting mental well-being among undergraduates.

### 4.2 LPA isochronous substitution for sedentary behavior, sleep, and MVPA reduces StD risk

This study found that substitutions of 5–10 min of LPA effectively reduced the risk of StD, suggesting that undergraduates could benefit from integrating brief activity bouts during breaks between classes. The depression-reducing effects of isochronous substitution are expected to accumulate over time (Table 4). Notably, moderate to vigorous physical activity (MVPA) was associated with increased depression scores, which contrasts with existing findings that generally favor its depression-reducing effects (Roeh et al., 2020).

There are several possible explanations for these findings. First, the average levels of PA across all intensities in this study exceeded those reported in previous research and guidelines, reflecting a U-shaped relationship between exercise and depression scores (Kim et al., 2018). This finding aligns with Pearce’s findings, which suggest that excessive PA may diminish its benefits and increase uncertainty in its effects (Pearce et al., 2022). While PA generally has the potential to alleviate StD, excessive PA may exacerbate it. Therefore, LPA may be the optimal intensity for managing subclinical depression, as supported by recommendations from prior research (Pasanen et al., 2022). Modifications in the PA composition, particularly the LPA to MVPA ratio, could be considered in future interventions, potentially fostering sustainable PA habits among undergraduates.

Moreover, individuals who have higher levels of comorbid state anxiety along with depression may derive fewer benefits from depression-reducing exercise interventions (Blumenthal et al., 2021), indicating that supplementary strategies targeting anxiety may be necessary. Furthermore, if vigorous physical activity (VPA) exceeds the acceptable threshold for undergraduates accustomed to prolonged sedentary behavior, it could have counterproductive effects (Zahrt and Crum, 2020). Therefore, tailored interventions and PA prescriptions that consider individual characteristics and preferences are crucial for effectively addressing mental health challenges in this demographic.

## 5 Summary

This study comprehensively evaluated the impact of physical activity, sleep, and sedentary behavior on subthreshold depression among undergraduates, identifying critical risk factors and demonstrating the potential of activity substitution in depression regulation. Sedentary time exceeding 12 h and a PSQI score of 3.5 or higher were associated with an increased risk of subclinical depression. However, this study has certain limitations that must be acknowledged.

First, the research was conducted within a single academic institution, which may limit the generalizability of the findings. The cut-off values and the effects of isochronous substitution may vary across different populations, so future studies should include more diverse cohorts to enhance the representativeness of the results. Second, the reliance on self-reported questionnaires rather than objective measurements may introduce potential biases, including recall bias. Therefore, future research should consider employing multicenter studies or integrating evidence-based approaches to enhance the accuracy and applicability of mental health promotion and intervention strategies within educational settings.

## Data availability statement

The datasets presented in this article are not readily available because the data collection is ongoing and can only be used for studies related to depression in undergraduate students. Requests to access the datasets should be directed to 1132083877@qq.com.

## Ethics statement

The studies involving humans were approved by the Ethics Committee of Southern Medical University. The studies were conducted in accordance with the local legislation and institutional requirements. The participants provided their written informed consent to participate in this study.

## Author contributions

YM: Formal analysis, Funding acquisition, Methodology, Writing – original draft. YG: Writing – review & editing. HY: Investigation, Software, Writing – original draft. YZ: Investigation, Software, Writing – original draft. YK: Writing – review & editing, Methodology.
